# Extrusion of the anterior segment of the medial meniscus extrusion initiates knee osteoarthritis: evaluation using magnetic resonance imaging

**DOI:** 10.1186/s40634-023-00693-x

**Published:** 2023-12-13

**Authors:** Atsushi Ichiba, Eichi Ito, Keiichiro Kino

**Affiliations:** Department of Orthopaedic Surgery, Shinkawabata Hospital, 2-31-1, Ichimonbashi, Nagaokakyo city, Kyoto, 617-0825 Japan

**Keywords:** Medial meniscus, Extrusion, Osteoarthritis, Magnetic resonance imaging

## Abstract

**Purpose:**

Meniscus extrusion contributes to the progression of knee osteoarthritis (OA). It is not clear which site of the medial meniscus (MM) extrusion (MME) is greatest. Moreover, the relationship between sites of MME and progression of OA has not yet been elucidated. The purpose of this study was to determine which sites of MME that showed the greatest extrusion and to investigate the relationship between the presence of MM tears and MME, the relationship between the progression of OA and MME.

**Methods:**

A cohort of 111 patients were studied retrospectively. The OA grade was classified using the Kellgren-Lawrence (K-L) grade. MME was measured at 13 positions from the anterior to the posterior segment using magnetic resonance imaging (MRI) with slices perpendicular to the MM (radial MRI). The relationship between the K-L grade and the site of the MME was investigated. The patients were grouped as follows: The patients over 40-years-old were grouped as follows: patients with the K-L grade ≤1 and without a MM tear (Group En (early, no meniscus tear)); patients with the K-L grade ≤1 with a MM tear (Group Ep (early, positive meniscus tear)); patients with the K-L grade ≥2 and without a MM tear (Group An (advanced, no meniscal tear)); patients over-40 years-old with the K-L grade ≥2 and with a MM tear (Group Ap (advanced, positive meniscus tear)). And patients between 15 and 39-years-old with no abnormal findings on MRI were defined as control group (Group C).

**Results:**

In the Groups En and Ep, MME was greatest in the anterior segment, and was greater in Group Ep than in Group En. In Groups Ap and Group C, extrusion was greatest in the middle segment.

**Conclusion:**

The results suggest that MME predominantly occurred in the anterior segment with increasing age, after that, MM extruded at the middle segment with progression of OA and MM tear.

Level of evidence IV

**Supplementary Information:**

The online version contains supplementary material available at 10.1186/s40634-023-00693-x.

## Introduction

Medial meniscus (MM) extrusion (MME) is considered to result from the disruption of circumferential collagen fibers, which causes the loss of the ability to resist hoop strain [25]. Extruded MM can increase the peak contact pressure in the medial compartment of the knee, similar to total meniscectomy, which can lead to arthritic changes [23]. An MME ≥3 mm is considered an abnormal pathologic extrusion that correlates with the severity of chondral lesions in the medial compartment [8, 11, 29]. MME is known to occur in meniscus tears [8, 12, 15, 16, 22, 30], and is one of the known causes of osteoarthritis (OA) progression [4–6, 8–13, 16–19, 27, 30]

Most existing studies on MME, measure extrusion at the middle segment on a coronal slice using magnetic resonance imaging (MRI) [7, 11, 29]. However, when evaluating MME using MRI coronal slices, a cross section perpendicular to the MM can be created only near the center of the anterior-posterior diameter of the meniscus, whereas in other slices, a cross-section is created obliquely against the meniscus and the exact amount of extrusion is unknown. Few studies have been conducted on the site of meniscal extrusion. Some researchers have reported that MME is greater in the anterior horn [4, 5, 9, 19, 28]. In their reports, extrusions of the anterior and posterior horns were measured on a sagittal slice on MRI, and extrusion of the middle segment was measured on a midcoronal slice. However, significance of the extrusion of the anterior horn being greater than that of the middle segment has not been discussed.

Abnormalities in the attachment of the anterior horn of the MM have been reported as factors influencing OA progression [3, 26]. If the anterior horn attachment of the MM is located on the anterior slope of the proximal tibia or does not have bony attachment, the meniscus may not be able to resist load stress and may be prone to extrusion [26]. In this case, extrusion at the anterior segment is predicted to greater than that at the middle segment.

Based on these reports, we hypothesized that MME of the anterior segment would be greater in patients with knee OA. In this study, we circumferentially investigated the MME on MRI with slices perpendicular to the MM (radial MRI) to determine where extrusion was greatest, and whether the site　of MME contributes to the progression of OA. Therefore, we aimed to determine which sites of MME showed the greatest extrusion. Additionally, we aimed to investigate the relationship between the presence of MM tears and MME, the relationship between the progression of OA and MME, and the factors that affect MME. There have been no studies investigating the relationship between the site of MME and the progression of OA.

## Materials and methods

### Study participants

The study included consecutive patients with knee joint symptoms who visited our outpatient orthopedic clinic between June, 2021 and June, 2022. Radiographs and unilateral radial MRI of the knee joint were performed for each patient. This study was conducted retrospectively. Tumors, dislocations, fractures, ligament injuries, pediatric cases (age <15 years old), cases of valgus knee deformity (femorotibial angle (FTA) <170°), and cases with a body mass index (BMI) >30 kg/m^2^ were excluded. Patients in whom MM was unreadable on MRI were also excluded.

### Radiograph assessments

For each patient, a series of knee radiographs (standing anteroposterior, lateral, and skyline radiographs) were obtained. OA grade was classified using the Kellgren-Lawrence (K-L) grading system [20]. In this study, a K-L grade ≥2 was considered to indicate of radiographic OA. The anatomical FTA were measured. All radiographic images were digitally acquired using a picture archiving and communication system (PACS) (Rapideye, Toshiba Medical Systems, Tochigi, Japan), and assessments were performed using PACS software.

### MRI assessment

MRI was performed using a Vantage XGV 1.5 T (Toshiba Medical Systems, Tochigi, Japan) with a knee coil in the knee extension position. Standard sequences included the following: proton density coronal (TR/TE: 2200/15), sagittal T1-weighted (TR/TE: 465/12), coronal T2* (repetition time [TR]/echo time [TE]: 530/15), sagittal T2* (TR/TE: 530/15) and radial T2* (TR/TE: 560/15).

Measurement of medial meniscus extrusion in radial sections

**Fig. 1:**
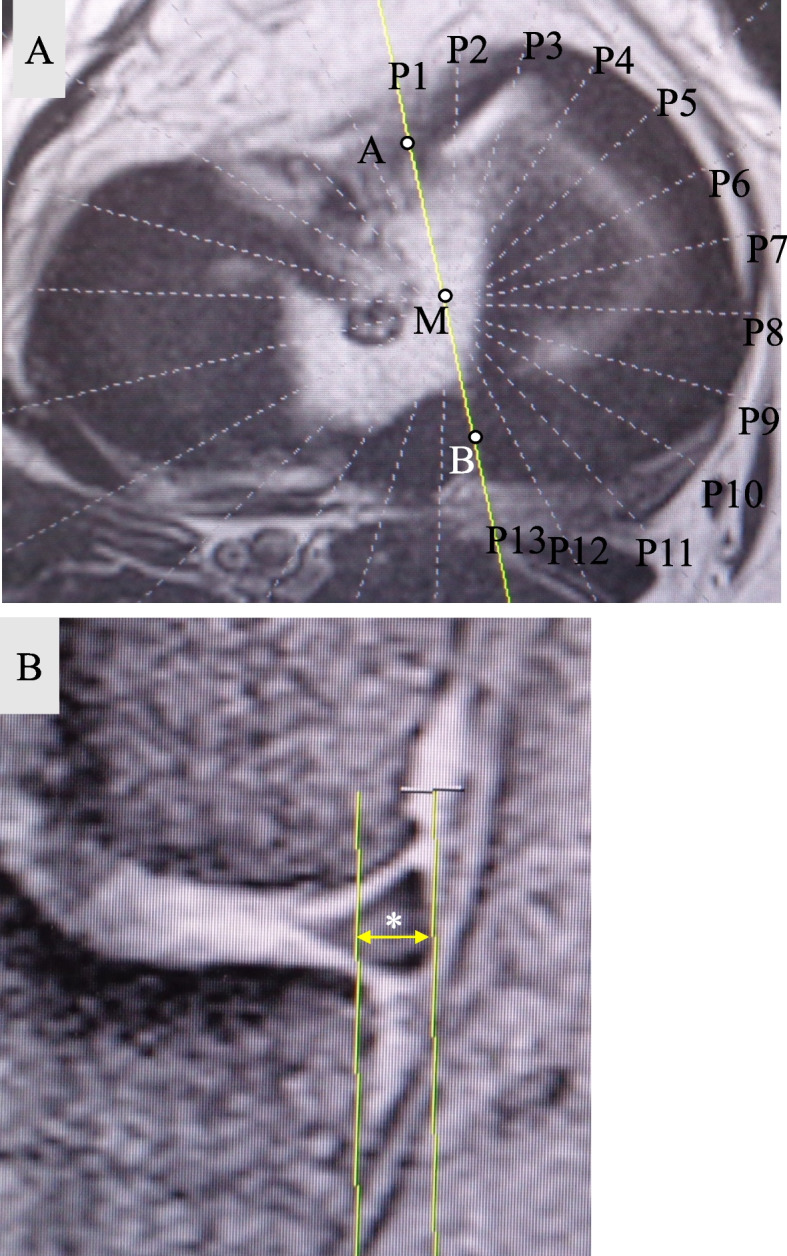
**A** Schematic illustration of slice selection on magnetic resonance imaging. Point A; Point at the center of the anteroposterior diameter of the anterior horn of the medial meniscus. Point B; Point at the center of the anteroposterior diameter of the posterior horn of the medial meniscus. Point M, the midpoint of line AB was designated as point M. Slice position 1 was represented as P1. **B** In each slice, MME from the tibial articular surface, excluding the osteophytes, was measured (*). The displacement of the medial meniscus from the margin of the medial tibial plateau was measured. A vertical line was drawn intersecting the outer margin of the medial tibial plateau. Medial meniscus extrusion was measured from this line to the outer edge of the medial meniscus

Figure 1A shows the method for determining the slice position on MRI. An axial section of the MM was obtained on a T2* image. The center of the anteroposterior diameter of the anterior horn of the MM was designated as point A, and the center of the anteroposterior diameter of the posterior horn was designed as point B. A line AB drawn and its midpoint set as point M. Twelve slices were made radially to the MM at 15° each, centered on point M. Slice positions 1–4 was defined as the anterior segment, positions 5–9 as the middle segment, and positions 10–13 as the posterior segment. Slice position 1 is represented as P1 in this study.

In each slice, MME from the tibial articular surface, excluding the osteophytes, was measured (Fig. 1B). The displacement of the MM from the margin of the medial tibial plateau was measured. A vertical line was drawn intersecting the outer margin of the medial tibial plateau. MME was measured from this line to the outer edge of the MM. The relative error of MME length measurement on the DICOM system was 0.5%.

### Evaluation of the medial meniscus tear

Meniscal damage was assessed using the whole-organ magnetic resonance score (WORMS), grading from 0 to 4 [24]. This evaluation was performed in coronal and sagittal views. The anterior horn, middle segment, and posterior horn of the MM were graded from 0 to 4 based on both sagittal and coronal images; with 0 = intact menisci, 1 = minor radial tear or parrot-beak tear, 2 = non-displaced tear or prior surgical repair, 3 = displaced tear or partial resection, and 4 = complete maceration/destruction or complete resection. A tear was defined by a signal change within the meniscus that extended to the surface of the meniscus.

### Subgroup analysis

Patients with a K-L grade 0 or 1 and those ≥40 years of age were defined as Group E (early). Patients with K-L grades 2, 3, and 4 and aged ≥40 years were classified into Group A (advanced). Patients aged between 15 and 39 years whose MRI results did not show any obvious abnormalities were defined as the control group (Group C). We divided our analysis into subgroups to investigate the association between meniscal tears, OA changes, and MME, as follows: within Group E, cases without MM tears were designated as Group En (early, no meniscus tear), and cases with MM tears were designated as Group Ep (early, positive meniscus tear). Within Group A, cases without MM tears were designated as Group An (advanced, no meniscal tear), and cases with MM tears were designated as Group Ap (advanced, positive meniscus tear).

### Evaluations

We evaluated the MME of 13 slice positions measured on the radial MRI. Second, the sites with the maximum MME were identified. The position with the highest MME value from P1 to P13 was defined as the maximum MME for the knee. Third, the relationship between the development of OA (K-L grade) and MME was evaluated, along with the relationship between the presence of MM tears and MME, and factors that affected MME were investigated. For this analysis, the total MME in the anterior and middle segments (P1–9) was defined as the amount of MME in the knee.

### Reproducibility measurements

Two orthopedic surgeons conducted the examination to assess interobserver reproducibility (A.I, E.I.). The kappa coefficient showed that the interobserver reliability for the K-L grade was 0.93 (95% confidence interval (CI): 0.84–1.0), and WORMS was 0.91 (95% CI: 0.80–0.99). MME was independently measured by two orthopedic surgeons (A.I, E.I.). To calculate the interobserver reproducibility, five randomly selected cases were read again by the same two readers, at an interval of 1 month between readings and assessed by an interclass coefficient (ICC). The intraobserver reproducibility of the MME measurement was 0.95 (95% CI: 0.94–0.98), the interobserver reproducibility was 0.88 (95% CI: 0.82–0.93). Additionally, we performed Bland-Altman analysis in interobserver reliability analysis and found good reliability at all positions from P1 to P13.

### Statistical analysis

Data are presented as the mean ± standard deviation. One-way analysis of variance (ANOVA) with Tukey’s test was used to compare the demographic data and MME. Analysis of co-variance (ANCOVA) was used for further confounding analysis if needed. Pearson’s correlation coefficient was used to test for correlations. The chi-squared test was used for categorical variables. The factors affected MME were analyzed using multiple regression or binary logistic analysis. Statistical significance was set at *P* <0.05. All statistical analyses are performed using Bell Curve for Excel (Social Survey Research Information. Co., Ltd., Shinjuku, Tokyo, Japan). Before the study, a sample size analysis focusing on MME was performed using G* power 3.0 (Dusseldorf, Germany). To achieve 80% statistical power with an *α* error of 0.05 in demonstrating large effect size (*f* = 0.4), power analysis revealed that a total sample size of 76 patients would be required for detecting differences in MME with ANOVA.

## Results

### Demographics

**Fig. 2:**
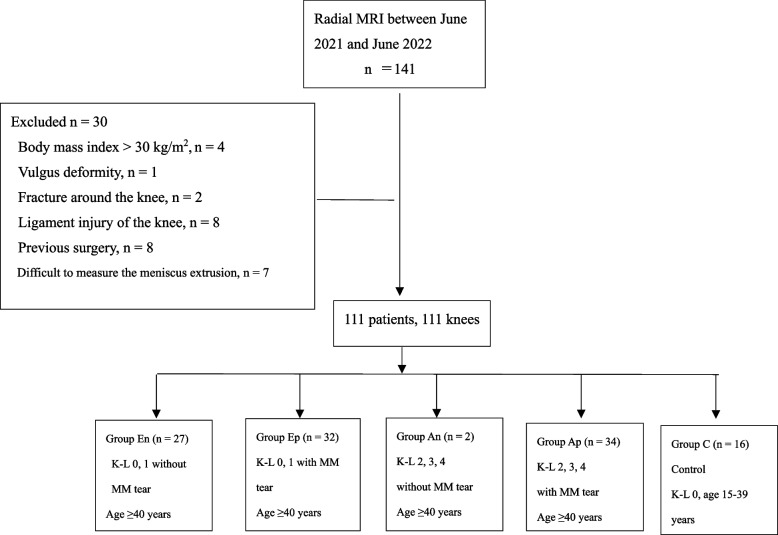
Flowchart summarizing the subject inclusion/exclusion process. MRI, magnetic resonance imaging; n, numbers; K-L, Kellgren-Lawrence grade; MM, medial meniscus; Group En, non- or early osteoarthritis without meniscus tear; Group Ep, non- or early osteoarthritis with meniscus tear; Group An, advanced osteoarthritis and without meniscus tear; Group Ap, advanced osteoarthritis and with meniscus tear; Group C, control group

A total of 111 patients (111 knees) who met the inclusion criteria were included in the final study cohort, a flowchart of the patient selection is shown in Fig. 2. They consisted of 51 males and 60 females with a mean age of 57.0 ± 17.7 years (range, 15–84 years), mean BMI of 22.8 ± 3.0 kg/m^2^ (range, 16.1–29.1 kg/m^2^), mean FTA of 177.8 ± 2.9° (range, 173.5–184.9°). The demographic data of the subgroups are presented in Table 1. Group An was excluded from the statistical analysis because it contained only two cases. The mean age of the patients in Group Ep was significantly higher than that of the patients in Group En. The FTA in Group Ap was significantly greater than that in Group Ep.

**Table 1:** Demographic data of participants in subgroups

	Group E		Group A		Group C	Statistics
	Group En	Group Ep	Group An	Group Ap		
No. of patient/knees	27/27	32/32	2/2	34/34	16/16	
Sex, men/women, n^a^	11/16	15/17	0/2	17/17	8/8	n. s. ^e^
Age, years (SD^b^) Range	55.4 (12.3)	64.3 (11.9)	64.5 (10.6)	66.3 (10.5)	22.0 (3.1)	f
	41–82	43–84	50–56	51–82	15–39	
Body mass index, kg/m^2^ (SD ^b^), range	21.9 (3.0)	23.5 (9.4)	22.3 (2.1)	22.0 (3.1)	21.9 (3.1)	n. s. ^e^
	16.9–29.1	20.0–29.0	20.8–23.8	19.1–29.0	16.6–28.9	
Side, right /left, n^a^	16/11	14/18	1/1	18/16	10/8	n. s. ^e^
Kellgren-Lawrence grade Grade 0/1/2/3/4, n^a^	17/10/0/ 0/0	21/11/0/0/0	0/0/2/0/0	0/0/8/15/11	15/1/0/0/0	
Femorotibial angle (°^c^) (SD^b^), Range	177.9 (1.7)	177.6 (2.3)	175.9 (1.9)	179.4 (3.8)	176.9 (1.4)	g
	174.9–182.5	173.0–181.2	174.2–177.2	174.0–184.9	175.6–179.1	
Medial meniscus tear (WORMS^d^) Grade 0/1/2/3/4, n ^a^	27/0/0/0/0	0/2/16/8/6	2/0/0/0/0	0/0/5/9/20	16/0/0/0/0	
Lateral meniscus tear (WORMS^d^) Grade 0 /1/ 2/ 3/4, n^a^	25/1/0/0/0	29/1/2/0/0	2/0/0/0/0	29/3/0/1/0	16/0/0/0/0	

En vs. Ep, *P* = 0.015; En vs. Ap, *P* = 0.0017; En vs. C, *P* < 0.001; Ep vs. Ap, *P* = 0.90; Ep vs. C, *P* < 0.001. g, Results of statistical analysis: En vs. Ep, *P* = 0.23; En vs. Ap, *P* = 0.13; En vs. C, *P* = 0.57; Ep vs. Ap, *P* < 0.001; Ep vs. C, *P* = 0.99.

Group E, non- or early osteoarthritis group; Group A, advanced osteoarthritis group; Group C, control group; Group En, non- or early osteoarthritis without meniscus tear; Group Ep, non- or early osteoarthritis with meniscus tear; Group An, advanced osteoarthritis and without meniscus tear; Group Ap; advanced osteoarthritis and with meniscus tear.

The number of patients in group An was two, thus statistical analysis was not performed

^a^n, numbers

^b^SD, Standard deviation

^c^^°^, degrees

^d^WORMS, Whole-Organ Magnetic Resonance Score

^e^n.s., not significant

^f^Results of statistical analysis

### Comparison of Group En and Group Ep

**Fig. 3:**
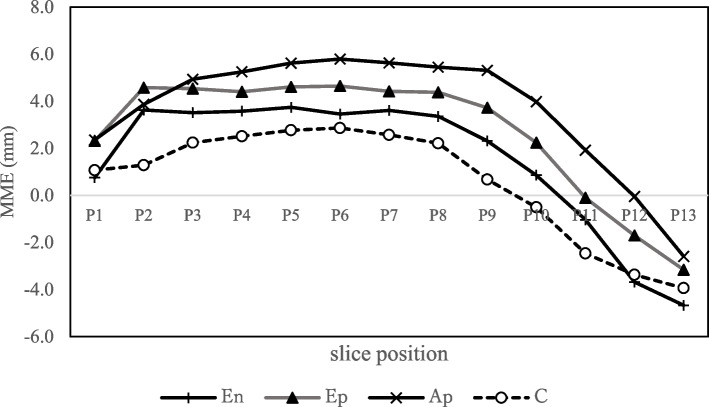
Medial meniscus extrusion in the subgroups. En, Group En, non- or early osteoarthritis without meniscus tear; Ep, Group Ep, non- or early osteoarthritis with meniscus tear; Ap, Group Ap, advanced osteoarthritis and with meniscus tear; C, Group C, control group; MME, medial meniscus extrusion. P1, on radial MRI coronal slices were made radially from the anterior to posterior horns of medial meniscus, with the anterior horn slice as position 1 and the posterior horn slice as position 13

To investigate the effect of MM tears on MME, Groups En and Ep were compared. The mean MME was greater in both groups from the anterior to middle segments. The mean MME tended to be greater in Group Ep than in Group En, and was significantly greater at P1 and P6 (Table 2, Fig. 3). There was a significant difference in age between Groups En and Ep in the demographic data (Table 1). Therefore, we added an analysis by ANCOVA to determine whether MME was affected by age or MM tears. For analysis by ANCOVA, MME was assigned as the objective variable, meniscal tear as a fixed factor, and age as a covariate. The results showed that MM tears significantly affected all sites except P2, 10, and 11, while age significantly affected only P3 (Additional file 1: Appendix, Table A). This suggests that the presence of MM tears, rather than age, affects the MME.

**Table 2:** Medial meniscus extrusion in subgroups

	P1 ^a^	P2	P3	P4	P5	P6	P7	P8	P9	P10	P11	P12	P13
	Anterior segment				Middle segment					Posterior segment			
Group En													
Mean	0.8	3.6	3.5	3.6	3.7	3.4	3.6	3.4	2.3	0.9	−1.0	−3.7	−5.8
SD ^b^	1.9	2.2	1.4	1.4	1.4	1.4	0.8	1.3	1.6	1.9	2.5	2.6	1.0
Group Ep													
Mean	2.3	4.5	4.5	4.4	4.6	4.6	4.4	4.4	3.7	2.2	−0.1	−0.2	−2.8
SD ^b^	2.5	1.7	1.6	1.5	1.4	1.4	1.5	1.4	1.8	2.9	3.5	2.6	2.8
Group Ap													
Mean	2.4	3.5	4.9	5.2	5.6	5.8	5.6	5.4	5.3	3.9	1.9	−0.1	−3.0
SD ^b^	2.2	2.4	2.0	1.8	2.4	1.8	2.2	2.8	3.1	3.5	3.7	4.3	3.5
Group C													
Mean	1.1	1.3	2.3	2.5	2.8	2.9	2.6	2.2	0.7	−0.51	−2.5	−3.6	−3.9
SD^b^	2.1	1.8	1.9	1.5	1.5	1.1	1.2	1.7	2.0	2.7	2.8	2.5	2.2
En vs ^c^ Ep													
*P* value	**0.038**	0.31	0.12	0.18	0.26	**0.011**	0.21	0.18	0.086	0.25	0.68	0.11	0.18
Ep vs Ap													
*P* value	0.99	0.52	0.79	0.13	0.11	**0.0085**	**0.013**	0.13	**0.026**	0.068	0.058	0.21	0.85
En vs C													
*P* value	0.97	**0.0033**	0.099	0.14	0.32	0.56	0.17	0.24	0.10	0.43	0.49	0.99	0.84

Boldface text indicates statistical significance

Group E, non- or early osteoarthritis group; Group A, advanced osteoarthritis group; Group C, control group; Group En, non- or early osteoarthritis without meniscus tear; Group Ep, non- or early osteoarthritis with meniscus tear; Group An, advanced osteoarthritis and without meniscus tear; Group Ap, advanced osteoarthritis and with meniscus tear.

^a^P1, on radial MRI coronal slices were made radially from the anterior to posterior horns of medial meniscus, with the anterior horn slice as position 1 and the posterior horn slice as position 13

^b^SD, Standard deviation

^c^vs, versus

### Comparison of Group Ep and Group Ap

The effect of OA grade on MME was investigated by comparing the Groups Ep and Ap. In Group Ap, MME was greatest in the middle segment. A comparison of Group Ep and Group Ap showed a trend toward greater MME in Group Ap in all positions. At P6, P7, and P9, MME was significantly greater in group Ap (Table 2, Fig. 3). There was a significant difference in FTA between Group Ep and Group Ap in the demographic data (Table 1). Therefore, we performed ANCOVA to determine whether MME was affected by OA grade or FTA. For analysis using ANCOVA, MME was assigned as the objective variable, OA grade as a fixed factor, and FTA as a covariate. The results showed that OA grade was significantly affected at P6 only, whereas FTA significantly affected P4, 5, 9, and 13. This suggests that the FTA affected the MME more than the OA grade (Additional file 1: Appendix, Table B). However, in the Ep and Ap groups, K-L grade and FTA were positively correlated (*r* = 0.425, *P* <0.001), suggesting that the progression of OA affected the MME.

### Comparison of Group En and Group C

The effect of age on MME was investigated by comparing the En and C groups. In Group C, the MME was greatest in the middle segment. The mean MME was greater in Group En than in Group C, and at P2, the MME was significantly greater in Group En (Table 2).

### Sites of maximum MME

**Fig. 4:**
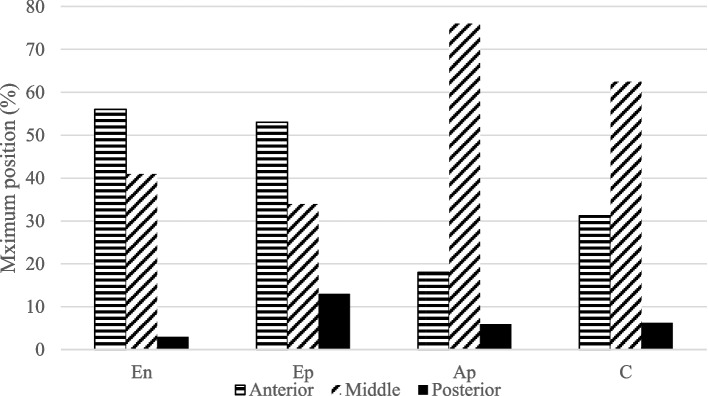
Results of maximum position of the medial mensicus extrusion. En, Group En, non- or early osteoarthritis without meniscus tear; Ep, Group Ep, non- or early osteoarthritis with meniscus tear; Ap, Group Ap, advanced osteoarthritis and with meniscus tear; C, Group C, control group; Anterior, anterior segment of the medial meniscus; Middle, middle segment of the medial meniscus; Posterior, posterior segment of the medial meniscus

The results for sites with the maximum MME are shown in Fig. 4. In Groups En and Ep, MME was evident in the anterior segment. In contrast, in Groups Ap and C, MME was evident in the middle segment (chi-square test, *P* = 0.012).

### Factors which affected MME

The statistical analysis of the factors affecting the MME is presented in Table 3. Age, K-L grade, and the presence of MM tears were shown to affect MME.

**Table 3:** Analysis of factors affecting the medial meniscus extrusion

Multiple linear regression analysis						
Dependent variables	Independent Variables	Non-standardized coefficients		Standardized coefficients		
		B	SE	β	*P* value	
Medial meniscus extrusion	Age	0.17	0.072	0.21	**0.018**	
	Body mass index	0.13	0.27	0.040	0.62	
	K-L grade	4.68	0.85	0.48	**< 0.001**	
	Femorotibial angle	− 0.0050	0.0072	− 0.055	0.49	
Logistic regression analysis						
Dependent variables	Independent variables	*P* value	Odds ratio	95% Confidence Interval		Standard Error
				Lower	Upper	
Medial meniscus extrusion	Sex	0.21	0.98	0.96	1.01	0.014
	Medial meniscus tear	**< 0.001**	1.16	1.09	1.23	0.031

*K-L grade* Kellgren-Lawrence grade, *B* Partial regression coefficient, *SE* Standard error, *β* Standard partial regression coefficient

Boldface text indicates statistical significance

## Discussion

The most important finding of this study was that MME was greatest in the anterior segment in Group E (Groups En and Ep) and in the middle segment in Groups Ap and C. And also, the presence of meniscus tear, age-related changes, and progression of OA influenced the increase in MME.

Group E had the greatest MME in the anterior segment, with or without concomitant MM tears. Group Ep showed greater MME than Group En (Table 1 and Figs. 3 and 4), suggesting that MM tears resulted in increased MME but did not affect the site of extrusion in non-OA or early OA knees.

In contrast, most of the patients in Group A (Groups An and Ap) had MM tears, and the MME was greatest in the middle segment, according to the comparison results between Groups Ep and Ap (Table 2 and Figs. 3 and 4). This suggests that most cases of advanced OA complicate MM tear and that MME typically occurs in the middle segment.

The comparison between Groups En and C showed that age-related changes first appeared in the anterior segment (Table 2 and Figs. 3 and 4). This suggests that age-related changes affected MME and caused MME in the anterior segment. As such, we speculated that OA progression is initiated by the MME at the anterior segment with age, followed by extrusion at the middle segment or MM tear. This is the first study to demonstrate the relationship between age, OA progression, and sites of MME.

We hypothesized that MME would be greater in the anterior segment in patients with knee OA. However, our hypothesis was not confirmed, as the MME of the middle segment was greater in Group Ap. In Group Ap, the MME was presumed to increase in the middle segment due to the progression of OA, resulting in cartilage wear and progressive varus deformity.

It has been reported that abnormalities in the attachment of the anterior horn of the MM are associated with the progression of OA [3, 26]. This could be a reason that the MME at the anterior segment was greater in the Group E in our study.

Several researchers have reported MME of the anterior horn [2, 4, 5, 9, 19, 28]. Arepati et al. [2] investigated the association of anterior horn and middle segment extrusion with the degree of OA progression using MRI in 1191 patients. They reported that in advanced OA knees, extrusion of the anterior horn as well as extrusion of the middle segment was observed. Bloecker et al. [5] used three-dimensional MRI to measure extrusion of the meniscus in healthy subjects without OA with a mean age of 57 years (range, 45-79 years). The authors reported the greatest MME in the anterior horn. This corresponds to the results for group E in our study.

Voet et al. [28] measured MME in elderly subjects and reported the relationship between MME and OA progression. They reported that MME of the middle segment, but not the anterior horn, is significantly involved in OA progression. In their report, these patients were in the K-L grade 2 or higher group, corresponding to Group A in our study. The results of their study are in agreement with those of our study. Both the above-mentioned studies measured the MME of the anterior horn; the MME of the anterior segment was unknown. The anterior segment is anchored to the tibia by the coronary ligament, whereas the anterior horn is firmly anchored to the bone through the anterior root. Therefore, the anterior segment is considered to be more prone to extrusion.

Many reports have described the relationship between MME and meniscal tears [7, 11, 14–17, 27, 29, 31] by studying multiple factors related to extrusion, these factors include including, older age [1] , sex [6, 7, 31], BMI [9], history of knee trauma [9], bone marrow lesion [9], malalignment [9, 16], chondral lesion [4, 6, 8, 16, 26, 29] and OA [9, 12, 18, 19, 27, 29, 30]. Our study provides supporting evidence suggesting both meniscal tear and OA progression (Table 3).

Our study has several limitations. First, patients in the control group had no abnormal MRI findings, but were symptomatic. Second, the MME was measured as absolute values and was not expressed in proportion to individual body size [7, 26, 31]. Third, MRI was performed in the knee extension position. Thus, MRI of the knee in the flexion position or dynamic movement was not performed. It was also evaluated in the supine position without weight bearing. Because the meniscus has its functions during load bearing, the MME differs when measured under weightbearing conditions [1].

Finally, the influence of the type of meniscus tear was not examined.

One of the strengths of this study is that MME was measured circumferentially from the anterior to the posterior segment by MRI, which provides new insight into the progression of OA. Clinically, the results of this study showed that even if the patient is over 40 years of age and has a low grade of OA, extrusion of the anterior segment exists in many cases, so centralization [22] of the MM or osteotomy should be considered to prevent further progression of OA.

## Conclusion

In Group E, extrusion of the anterior segment was greater. Most cases in Group A are complicated by MM tears and have the greatest MME in the middle segment. In Group C, MME was also greatest in the middle segment. It was inferred that extrusion at the anterior segment occurred with age and as OA progressed, followed by extrusion at the middle segment and the MM tear. The presence of meniscus tear, age-related changes, and progression of OA influenced the increase in MME. This study provides new insights into the progression of OA by examining the site of meniscal extrusion using radial MRI.

### Supplementary Information

**Additional file 1: Table A.** Results of analysis of covariance in Group En and Group Ep. **Table B.** Results of analysis of covariance in Group Ep and Group Ap. Table A.

## Abbreviations

OA: Osteoarthritis
MM: Medial meniscus
MME: Extrusion of the medial meniscus
K-L: Kellgren-Lawrence
MRI: Magnetic resonance imaging
FTA: Femorotibial angle
BMI: Body mass index
