# Supplementary material for: Extrusion of the anterior segment of the medial meniscus extrusion initiates knee osteoarthritis: evaluation using magnetic resonance imaging
Source: J Exp Orthop. 2023 Dec 13;10:135. doi: 10.1186/s40634-023-00693-x (PMC10719179; doi:10.1186/s40634-023-00693-x)
Supplement: Supplementary file 1 — Additional file 1: Table A. Results of analysis of covariance in Group En and Group Ep. Table B. Results of analysis of covariance in Group Ep and Group Ap. Table A. [file 40634_2023_693_MOESM1_ESM.docx]

**Appendix**

**Table A** Results of analysis of covariance in Group En and Group Ep

|  | P1^a^ | P2 | P3 | P4 | P5 | P6 | P7 | P8 | P9 | P10 | P11 | P12 | P13 |
| --- | --- | --- | --- | --- | --- | --- | --- | --- | --- | --- | --- | --- | --- |
|  | Anterior segment | | | | Middle segment | | | | | Posterior segment | | | |
| MM  tear  P value | **0.017** | 0.065 | **0.0019** | **0.0078** | **0.0013** | **< 0.001** | **0.029** | **0.0073** | **0.0037** | 0.052 | 0.36 | **0.041** | **0.039** |
| Age  P value | 0.98 | 0.93 | **0.028** | 0.055 | 0.28 | 0.34 | 0.57 | 0.69 | 0.86 | 0.97 | 0.65 | 0.33 | 0.50 |

Boldface text indicates statistical significance.

MM, medial meniscus; ^a^ P1, on radial MRI coronal slices were made radially from the anterior to posterior horns of medial meniscus, with the anterior horn slice as position 1 and the posterior horn slice as position 13.

**Table B**

Results of analysis of covariance in Group Ep and Group Ap

|  | P1^a^ | P2 | P3 | P4 | P5 | P6 | P7 | P8 | P9 | P10 | P11 | P12 | P13 |
| --- | --- | --- | --- | --- | --- | --- | --- | --- | --- | --- | --- | --- | --- |
|  | Anterior segment | | | | Middle segment | | | | | Posterior segment | | | |
| OA  grade  P value | 0.75 | 0.064 | 0.81 | 0.48 | 0.44 | **0.047** | **0.062** | 0.37 | 0.17 | 0.16 | 0.094 | 0.36 | 0.90 |
| FTA  P value | 0.54 | 0.27 | 0.17 | **0.002** | **0.006** | 0.17 | 0.26 | **0.026** | **0.019** | 0.14 | 0.35 | 0.087 | **0.047** |

Boldface text indicates statistical significance.

^a^ P1, on radial MRI coronal slices were made radially from the anterior to posterior horns of medial meniscus, with the anterior horn slice as position 1 and the posterior horn slice as position 13.
